# Genetic demography at the leading edge of the distribution of a rabies virus vector

**DOI:** 10.1002/ece3.3087

**Published:** 2017-06-09

**Authors:** Antoinette J. Piaggio, Amy L. Russell, Ignacio A. Osorio, Alejandro Jiménez Ramírez, Justin W. Fischer, Jennifer L. Neuwald, Annie E. Tibbels, Luis Lecuona, Gary F. McCracken

**Affiliations:** ^1^ USDA/APHIS/WS/National Wildlife Research Centre Fort Collins CO USA; ^2^ Department of Biology Grand Valley State University Allendale MI USA; ^3^ Committee of Promotion and Animal Health Protection Rabies Campaign, San Luis Potosi City San Luis Potosi México; ^4^ Former State Chief of the National Campaign of Bovine Paralytic Rabies SENASICA/SAGARPA México City México; ^5^ Department of Biology Colorado State University Fort Collins CO USA; ^6^ USDA/APHIS/International Services Distrito Federal México; ^7^ Department of Ecology and Evolutionary Biology University of Tennessee Knoxville TN USA; ^8^ Present address: MVZ. Marcelino Champagnat 505 Colonia Guadalupe Tepatitlán de Morelos Jalisco Mexico

**Keywords:** *Desmodus rotundus*, genetic demography, leading edge model

## Abstract

The common vampire bat, *Desmodus rotundus*, ranges from South America into northern Mexico in North America. This sanguivorous species of bat feeds primarily on medium to large‐sized mammals and is known to rely on livestock as primary prey. Each year, there are hotspot areas of *D. rotundus*‐specific rabies virus outbreaks that lead to the deaths of livestock and economic losses. Based on incidental captures in our study area, which is an area of high cattle mortality from *D. rotundus* transmitted rabies, it appears that *D. rotundus* are being caught regularly in areas and elevations where they previously were thought to be uncommon. Our goal was to investigate demographic processes and genetic diversity at the north eastern edge of the range of *D. rotundus* in Mexico. We generated control region sequences (441 bp) and 12‐locus microsatellite genotypes for 602 individuals of *D. rotundus*. These data were analyzed using network analyses, Bayesian clustering approaches, and standard population genetic statistical analyses. Our results demonstrate panmixia across our sampling area with low genetic diversity, low population differentiation, loss of intermediate frequency alleles at microsatellite loci, and very low mtDNA haplotype diversity with all haplotypes being very closely related. Our study also revealed strong signals of population expansion. These results follow predictions from the leading‐edge model of expanding populations and supports conclusions from another study that climate change may allow this species to find suitable habitat within the U.S. border.

## 1 INTRODUCTION

Anthropogenic influences, including climate change, have and will continue to alter the geographic distributions of species that transmit pathogens of concern for wildlife, livestock, and humans (Bellard et al., 2013; Crowl, Crist, Parmenter, Belovsky, & Lugo, 2008; Kim et al., 2013; Rohr et al., 2011). Rabies virus (Genus *Lyssavirus*) causes fatal disease in mammals, affecting wildlife, livestock, and humans globally. As carnivore rabies has come under greater control, rabies in Latin America now is transmitted to humans and livestock principally by common vampire bats (*Desmodus rotundus*; Geoffroy 1810) (Schneider et al., 2009; Velasco‐Villa et al., in press). It is estimated that the rabies virus entered bats about 700 years ago (Kuzmina et al., 2013; Smith, Orciari, & Yager, 1995) where it has since evolved into unique viral lineages within many bat species, including *D. rotundus* (Streicker et al., 2010). Significant economic damage is caused by *D. rotundus* rabies virus variants through loss of livestock throughout Latin America and the need for prophylaxis in human and domestic animals (Acha, 1967; Acha & Malaga‐Alba, 1988; Anderson et al., 2014; see review in Streicker et al., 2010). Thus, any range expansions of rabies virus vectors, particularly *D. rotundus*, could lead to increased transmission of the disease and subsequent economic impacts (Anderson et al., 2014).

*Desmodus rotundus* ranges throughout most of South and Central America and into northern Mexico in North America (Greenhall, Joermann, & Schmidt, 1983; Wilson & Reeder, 2005). Within the past 5 years, *D. rotundus* has been recorded within 50 km of the United States (U.S.) border in the Mexican state of Tamaulipas (Méxican Secretariat of Agriculture, Livestock, Rural Development, Fisheries, and Food (SAGARPA), unpublished data), which is north of their documented range limit. The fossil record shows that between 30,000 and 5,000 years before present (yBP), *D. rotundus* ranged within the current borders of the United States (Ray, Linares, & Morgan, 1988). Fossil records also indicate the presence of multiple *Desmodus* species (*D. stocki*,* D. archaeodaptes*,* D*. *draculae*, and *D. rotundus*) from deposits in present‐day New Mexico, Arizona, California, Texas, Florida, and West Virginia (Czaplewski & Peachey, 2003; Ray et al., 1988). It has been suggested that the northern distribution of *D. rotundus* is limited by the 10°C January minimal isotherm and that temperature and the bats’ rate of metabolism are correlated (Lee, Papes, & Van Den Bussche, 2012; McNab, 1973). The 10°C minimal isotherm corresponds to areas in the northern hemisphere that, on average, experience minimum daily temperatures of 10°C during the month of January. It is likely that climate change will push the 10°C minimal isotherm northward and thus shift the northern limits of the *D. rotundus* distribution (Walther et al., 2002).

As described under the leading‐edge model (Hewitt, 1993, 1996, 1999, 2000), populations that have expanded rapidly from a core population are expected to experience a loss of genetic diversity due to genetic drift operating through repeated bottlenecks or founder events. Under this model of range expansion, population expansion occurs through long‐distance dispersal and subsequent exponential population growth (Hewitt, 1999, 2000). Such populations may have relatively little variation within the region of population expansion (Hewitt, 2000; Ibrahim, Nichols, & Hewitt, 1996). This model characterizes genetic diversity of many temperate species populations that expanded into newly suitable habitat after the retreat of glaciers (e.g., Arbogast, 1999; Lessa, Cook, & Patton, 2003; Stone & Cook, 2000). Thus, if population genetic studies of *D. rotundus* on the northern edge of their distribution show low levels of genetic diversity and evidence of recent demographic and spatial expansion, then *D. rotundus* may be expanding their range.

Phylogeographic studies based on mitochondrial cytochrome *b* sequences have examined samples from core portions of the *D. rotundus* range (Brazil, Ditchfield, 2000; Brazil, French Guyana, and Costa Rica, Martins, Ditchfield, Meyer, & Morgante, 2007; southern Mexico to eastern South America/southern Brazil, Martins, Templeton, Pavan, Kohlbach, & Morgante, 2009), revealing high genetic diversity and differentiation among clades associated with different ecoregions dating to the Pleistocene, but no significant differences among roosts within ecoregions (Ditchfield, 2000; Martins et al., 2007, 2009). Some of these studies conclude that based on the distribution of genetic diversity across the sampled range of *D. rotundus*, that this species may have existed as isolated, refugial populations in South America during the Pleistocene (Martins et al., 2007, 2009). However, none of these studies (Ditchfield, 2000; Martins et al., 2007, 2009) included samples from central or northern Mexico. A recent population genetic study of *D. rotundus* throughout Mexico detected significant population structure among 15 sampling sites, but did not test for population expansion (Romero‐Nava, León‐Paniagua, & Ortega, 2014).

In another study using samples from this edge *D. rotundus*’ range, we used species distribution modeling with 7,094 capture records and five approaches and then paired with 17 climate change models to generate one ensemble prediction map for suitable habitat. The results suggest that climate change could result in suitable habitat for the expansion of *D. rotundus* into the southern tip of Texas or across Cuba into Florida (Hayes & Piaggio in review). Recent incidental records of *D. rotundus* within 50 km of the Mexico/U.S. border and apparent increased captures in areas and elevations where *D. rotundus* was thought to be uncommon (personal communications of local producers in Tamaulipas State) suggest that this process may be in progress. The potential establishment of populations in the US is of concern given that a rabies epidemic in livestock (vampire‐bat variant) has expanded from the southeast to northwest across the states of San Luis Potosí and Tamaulípas, Mexico since 2001 (SAGARPA). Population genetic analyses can be used to test predictions from the leading‐edge range expansion model and provide an additional test of the potential for *D. rotundus* to expand its geographic distribution across the Mexico/U.S. border.

In this study, we use population genetic approaches to examine populations of *D. rotundus* at the north eastern edge of the species’ distribution. We evaluate signals of demographic and spatial population expansion and levels of genetic diversity. We assessed demographic events with more rapidly evolving markers than used in previous studies of *D. rotundus* (Ditchfield, 2000; Martins et al., 2007, 2009), by investigating sequence variation in the mitochondrial DNA (mtDNA) hyper‐variable I segment of the control region (HV1) and at 12 nuclear microsatellite loci (Piaggio, Johnston, & Perkins, 2008). If the leading‐edge model accurately describes *D. rotundus* populations at the north eastern edge of its range, then we will find low genetic diversity and signals of recent population expansion.

## 2 MATERIALS AND METHODS

### 2.1 Sample collection and DNA extraction

**Figure 1:**
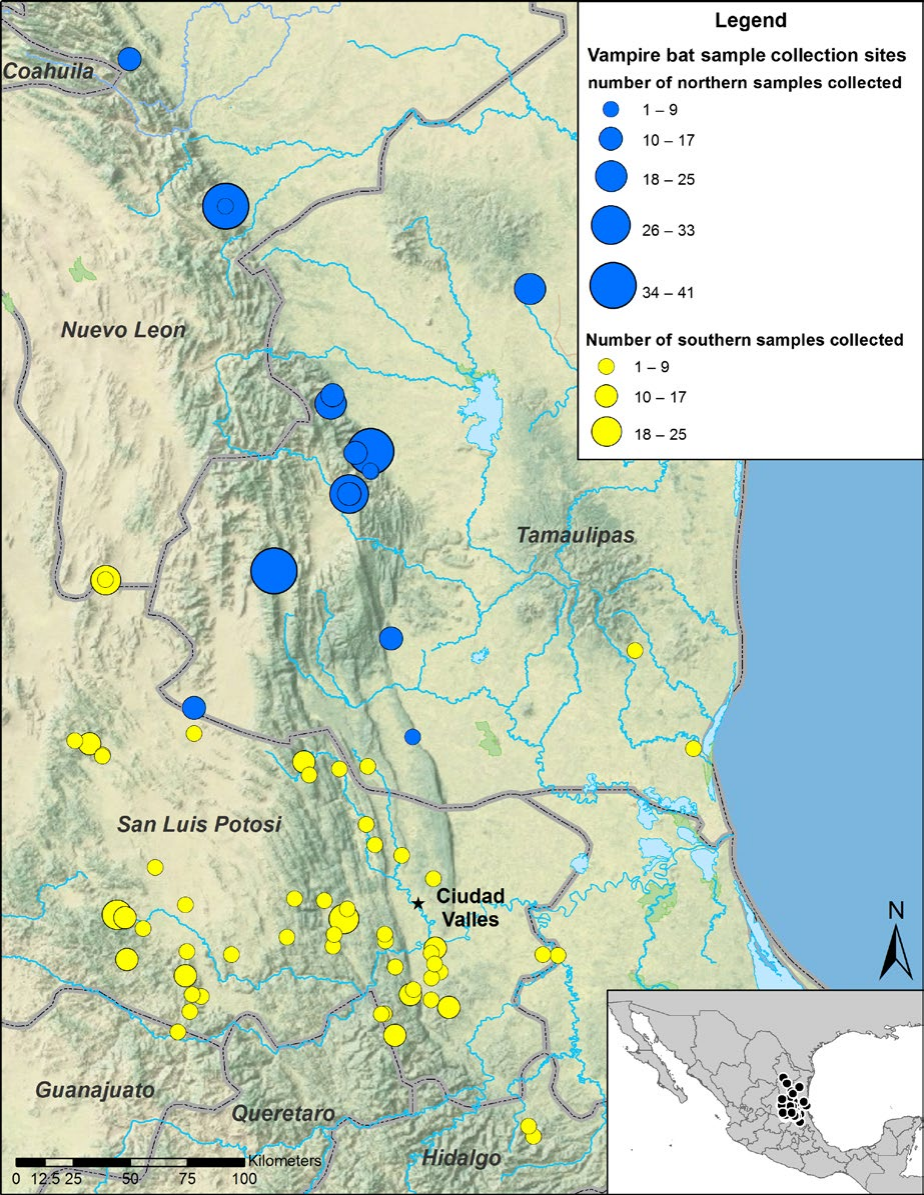
Map of sampling area. Colors denote two separate sampling efforts, the size of the circle indicates sampling effort at that site. In blue, samples were collected from 2003 to 2005, samples were from the states of Tamaulípas, and Nuevo Leon were obtained as wing biopsies from bats captured using mist nets, harp traps, or hand‐held nets at roosts and corrals (*n *=* *288). In the second sampling effort (yellow), tissue samples were obtained between 2006 and 2010 during state and federal control efforts for *D. rotundus* in the states of San Luis Potosí and Tamaulípas (*n *=* *314). The black star indicates where samples from another study (Romero‐Nava et al., 2014) overlapped our sampling area; they collected 12 bats from Ciudad Valles, San Luis Potosí, Mexico

Materials for this study came from the combination of two independent sampling efforts (Figure 1). In 2003–2005, samples from the states of Tamaulípas and Nuevo Leon (Northern Samples in Figure 1) were obtained as wing biopsies from bats captured using mist nets, harp traps, or hand‐held nets at roosts and corrals (*n *=* *288). In the second sampling effort (Southern Samples in Figure 1), tissue samples were obtained between 2006 and 2010 during state and federal control efforts for *D. rotundus* in the states of San Luis Potosí and Tamaulípas (*n *=* *314). DNA from all samples was extracted using a DNeasy Blood and Tissue Kit (Qiagen, Germantown, Maryland USA).

### 2.2 DNA amplification, sequencing, and genotyping

We sequenced a portion of the mitochondrial HV1 using primers P* (Wilkinson, Mayer, Kerth, & Petri, 1997) and E, following Wilkinson and Chapman (1991). PCR‐amplified products were purified for sequencing using ExoSAP‐IT (Affymetrix Santa Clara, California, USA). Purified products (1 μL) were used in 10 μL cycle sequencing reactions for each primer (P* and E) with 1 μmol/L primer, 0.25 μL BigDye v3.1, and 2.275 μL 5× sequencing buffer (Life Technologies Carlsbad, California USA). Genotypes from 12 microsatellite loci were generated for individual *D. rotundus* following Piaggio et al. (2008). Sequences and genotypes were run on either an ABI 3130 or an ABI 3130xl genetic analyzer and visualized using genemapper v.4.0 software (Applied Biosystems Carlsbad, California USA). Sequences were reviewed, edited, and aligned with sequencher v.5.2 (Gene Codes Ann Arbor, Michigan USA). Microsatellite genotypes were converted from genemapper into genepop files for downstream conversion into appropriate input files for various analytical software through gmconvert (Faircloth, 2006).

### 2.3 Statistical analyses—DNA sequences

Network analyses were performed to assess relationships among *D. rotundus* mtDNA haplotypes using network v.4.613 (available at http://www.fluxus-engineering.com/sharenet.htm). The number of haplotypes was assessed, and a haplotype network was calculated using a median‐joining approach (Bandelt, Forster, & Röhl, 1999) with default settings including equal weights for transitions/transversions and ɛ = 0. divein (Deng et al., 2010) is an online, maximum‐likelihood tool that was used to calculate average sequence divergences among unique haplotypes from both the consensus and the most common haplotype. This program also was used to identify phylogenetically informative sites among sequences. In an expanding leading‐edge population, we expect to observe low genetic diversity (Parsons et al., 1997) and a starburst‐shaped haplotype network (Russell, Pinzari, Vonhof, Olival, & Bonaccorso, 2015) at a rapidly evolving locus such as HV1.

We used various approaches to test the hypothesis that leading‐edge populations harbor signals of population expansion in DNA sequences (Hewitt, 1999, 2000). The neutrality tests Fu's (1997) *F*_*S*_ and Tajima's (1989) *D* were used to test for population growth, with significance assessed via 10,000 simulated samples using arlequin v.3.5.1.2 (Excoffier & Lischer, 2010). A mismatch distribution was also generated in arlequin with 10,000 bootstrap replicates to test the hypothesis that polymorphisms among sequences fit a model of rapid demographic or spatial expansion (Rogers & Harpending, 1992; Slatkin & Hudson, 1991).

### 2.4 Statistical analyses—microsatellite genotypes

Using the entire dataset of 12‐locus genotypes for 602 *D. rotundus* individuals, null allele frequencies were calculated with a 95% confidence interval (Brookfield 1; Brookfield, 1996) using microchecker (Van Oosterhout, Hutchinson, Wills, & Shipley, 2004). arlequin v. 3.5.1.2 (Excoffier & Lischer, 2010) was used to calculate the number of alleles and gene diversity per locus, estimate average gene diversity across loci, and evaluate each locus for Hardy–Weinberg equilibrium (HWE, Nei, 1987). Tests of HWE were evaluated using Bonferroni's corrections for multiple tests (Rice, 1989). genalex v.6.5 (Peakall & Smouse, 2006) was used to examine diversity across loci, including the Information Index (*I*), which estimates diversity of alleles while accounting for evenness in the representation of those alleles across the samples and correcting for uneven sample sizes (Smouse, Whitehead, & Peakall, 2015). A low value of I suggests that a few alleles dominate while most are rare, which is expected during a population expansion/founder event or the emergence of cladogenesis (Smouse et al., 2015). fstat v.2.9.3.2 (Goudet, 1995) was used to calculate *F*_IS_ and assess genotypic disequilibrium among all individuals and across all loci.

Two tests of isolation by distance (IBD) were performed: one among roosts and another among individuals (Rousset, 2000). arlequin was also used to assess IBD using a Mantel test for correlation between Slatkin's linear *F*_ST_ and the log of the straight‐line distances among the 18 roosts where we caught ≥ 6 individuals. For this analysis, we considered only individuals captured at the entrance of or within roosts (*N *=* *394) rather than bats caught on the wing. A test of IBD by nonroost capture localities may pool individuals belonging to different populations and thus be susceptible to bias from the Wahlund (1928) effect, which would underestimate IBD. However, we were concerned that the IBD analysis between roosts may not adequately describe the relationships between genetic diversity and geographic distance across the sampling region due to our use of a subset of the total data, so we chose to also assess IBD across the entire sampling region and with all genotyped samples. IBD was calculated among all individuals (*N *=* *602) in pairwise comparisons. This test uses log‐transformed geographical distances, which are tested for correlation to probability of identity of genes between individuals (estimator â; Rousset, 2000). This analysis was performed using genepop v.4.2 on the Web (option 6; Raymond & Rousset, 1995; Rousset, 2008).

We used baps v.6.0 (Corander & Marttinen, 2006; Corander, Marttinen, Sirén, & Tang, 2008) to estimate the number of genetic clusters (*K*) distributed among samples, and among females separately, informed by spatial information about capture locality (Corander, Sirén, & Arjas, 2008). We assessed population structure among females alone to test for sex‐biased genetic clustering. The program was run as an assessment of spatial clustering of individuals with five replications of each value of *K* from 1 to 20. structure v.2.3.4 (Falush, Stephens, & Pritchard, 2003; Pritchard, Stephens, & Donnelly, 2000) also performs a Bayesian analysis to estimate genetic clustering but uses HWE as an optimization criterion, whereas baps seeks to construct genetically homogeneous clusters (François & Durand, 2010). structure was run with all *D. rotundus* genotypes and set with a burn‐in of 50,000 and 300,000 MCMC repetitions after burn‐in. The ancestry model was set to admixture, and allele frequencies were assumed to be correlated among individuals. Values of *K* from 1 to 20 were explored, with five replications of each *K* to assess the stability of results between runs (Waples & Gaggiotti, 2006).

We used a factorial correspondence analysis (FCA) implemented in genetix v.4.05.2 (Belkhir, Borsa, Chikhi, Raufaste, & Bonhomme, 1996) as another approach for visualizing how individuals are clustered based on shared alleles. FCA is similar to a principal components analysis as it is a multivariate ordination technique, but it uses categorical rather (alleles per locus) than continuous data to explore correspondence among individual genotypes. Widespread distribution and low correspondence of genotypes across three axes demonstrate a lack of population structure. Tight clustering of genotypes indicates low diversity, and multiple tight clusters show population differentiation. FCA differs from the Bayesian clustering methods in that it is model‐free and thus avoids any prior assumptions about the nature and the relationship of the data.

Population demographics were evaluated from microsatellite data by calculating the imbalance index β (Kimmel et al., 1998). This estimator (β = 1 stable populations; β > 1 recent expansion or recovery from previously reduced population; β < 1 recent expansion from stable population) is based on variance in repeat numbers and allele frequencies and was calculated with 95% confidence intervals using SAS package and a program written and shared by T. Lehmann (Donnelly, Licht, & Lehmann, 2001).

## 3 RESULTS

### 3.1 Genetic Diversity

**Figure 2:**
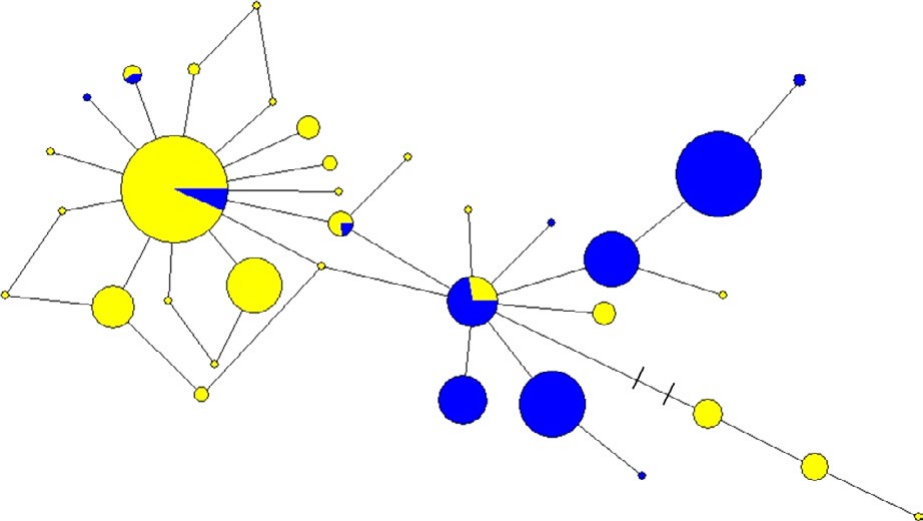
Haplotype network of HV1 sequences (441 bp) for 602 *D. rotundus* individuals. The 602 sequences comprised only 34 unique haplotypes, which all were closely related to each other. Hash marks show the only connection between haplotypes that represents a two base‐pair change, and all others are only a single base change. In blue, samples were collected from 2003 to 2005, samples were from the states of Tamaulípas and Nuevo Leon were obtained as wing biopsies from bats captured using mist nets, harp traps, or hand‐held nets at roosts and corrals (*n *=* *288). In the second sampling effort (yellow), tissue samples were obtained between 2006 and 2010 during state and federal control efforts for *D. rotundus* in the states of San Luis Potosí and Tamaulípas (*n *=* *314)

We generated HV1 sequences of 441 bp (GenBank Accession #‐) and 12‐locus genotypes for 602 *D. rotundus* individuals. The 602 sequences comprised only 34 unique haplotypes, which all were closely related to each other (Figure 2). Sequence divergences calculated from the most common haplotype and from the consensus sequence of all haplotypes were very low (both = 0.5%) with only 14 segregating sites among the unique haplotypes, which included 12 transitions and two single‐bp indels. Pairwise differences among these haplotypes as a proportion of sequence length ranged from 0.002 to 0.016, with an average of 0.007. The two sampling efforts combined for this study were largely distinct geographically (Figure 1) and temporally. However, all the haplotypes, from both studies, were closely related with limited geographical association evident (Figure 2).

Evidence of possible null alleles were detected at two microsatellite loci: Dero_G10F_B03R (frequency = 0.07) and Dero_H02F_C03R (frequency = 0.19). Because the frequency of potential null alleles was low (Dero_G10F_B03R) to moderate (Dero_H02F_C03R), we kept these loci in the analyses as they were not expected to bias our estimates because they occurred at < 0.20 (Dakin & Avise, 2004). Average gene diversity was moderate at 0.65 ± 0.33. The number of alleles across the 12 loci ranged from 2 to 23 per locus, and HWE was violated through excessive homozygosity at four loci (Table 1). The two loci with the biggest difference between H_O_ and H_E_ were the same two loci that demonstrated significant evidence of null alleles. The Information Index was low to moderate across loci (*I* = 0.56–2.57) with an average *I* = 1.37 ± 0.16, suggesting that most loci are characterized by a few very common alleles and more rare alleles. This pattern is evident when graphs of allele frequencies per locus are observed (Appendix S1). *F*_IS_ across all loci was 0.08 (*p *=* *0.05), which we interpret as being not biologically meaningful. No significant genotypic disequilibrium was detected in pairwise comparisons between all loci.

**Table 1:** The number of alleles per locus, observed heterozygosity (H_O_), expected heterozygosity (H_E_), and estimated null allele frequencies (Brookfield 1; Brookfield, 1996)

	# of alleles	H_O_	H_E_	NA
Dero_A08F_B01R	23	0.89	0.91	0.01
Dero_B03F_B03R	6	0.56	0.58	0.03
Dero_B10F_E01R	8	0.72^a^	0.75	0.02
Dero_B11F_B11R	3	0.48^a^	0.50	0.02
Dero_C07F_A02R	5	0.60	0.62	0.02
Dero_C11F_C11R	11	0.61	0.62	0.01
Dero_C12F_B02R	8	0.75	0.76	0.02
Dero_D02F_D02R	21	0.71	0.74	0.01
Dero_D06F_D06R	2	0.37	0.37	0.01
Dero_D12F_D12R	12	0.67	0.71	0.02
Dero_G10F_B03R	12	0.57^a^	0.69	0.07
Dero_H02F_C03R	7	0.25^a^	0.53	0.19

a Significant (*p* < 0.05) violations of HWE after Bonferroni's corrections (Rice, 1989). Two loci showed signs of null alleles; Dero_H02F_C03R had 7 alleles with two of them having allele frequencies of 0.93 together (101 = 0.33; 105 = 0.60). For locus Dero_G10F_B03R there were 12 alleles and allele frequencies for just two of the loci summed up to 0.75 (134 = 0.45; 144 = 0.30).

### 3.2 Population structure

**Figure 3:**
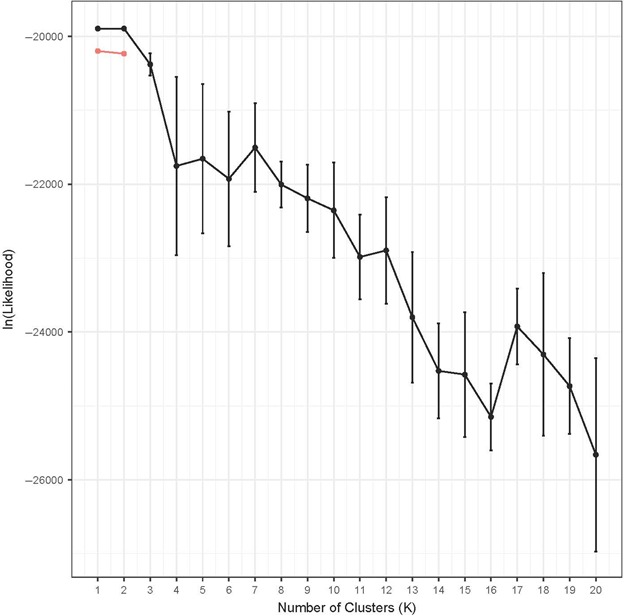
Log‐likelihood values (mean ± SD) for genetic clustering analyses of microsatellite data using structure. Log‐likelihood values (mean ± SD) for *K *=* *1 through *K *=* *10 from structure analyses are in black; those from the top 10 values across runs of *K* from baps, which was only *K *=* *1 and *K *=* *2 are in red

Microsatellite‐based tests of IBD considering genetic and geographic distance between roosts were significant (*r* ^2^ = 0.21; *p *<* *0.05). An analysis of IBD between individuals for the full dataset was highly significant (*p *<* *0.00001). Bayesian clustering analyses in both baps and structure provided the strongest support for a single genetic cluster (*K *=* *1) among all individuals (Figure 3). Although the average lnL scores for *K *=* *2 were not significantly different than for *K *=* *1 in structure results, baps showed that *K *=* *1 was the best explanation of genetic clustering across the samples. Furthermore, with two or more clusters, assignment of individuals often was apportioned into more than one cluster, and there was not geographical thus not biologically meaningful coherence. Separate analyses of females in baps also resulted in the most likely clustering as *K *=* *1. To test that the null alleles and subsequent HWE violations at Dero_G10F_B03R and Dero_H02F_C03R did not bias our results, we reran two analyses that assume HWE without these loci. First we estimated *F*_IS_ (0.03; *p *=* *0.05) and then we ran structure (*K *=* *1, average lnL = −16974.7; *K *=* *2, average lnL = −17177.4), which showed stronger support for a single cluster after removing loci with null alleles.

**Figure 4:**
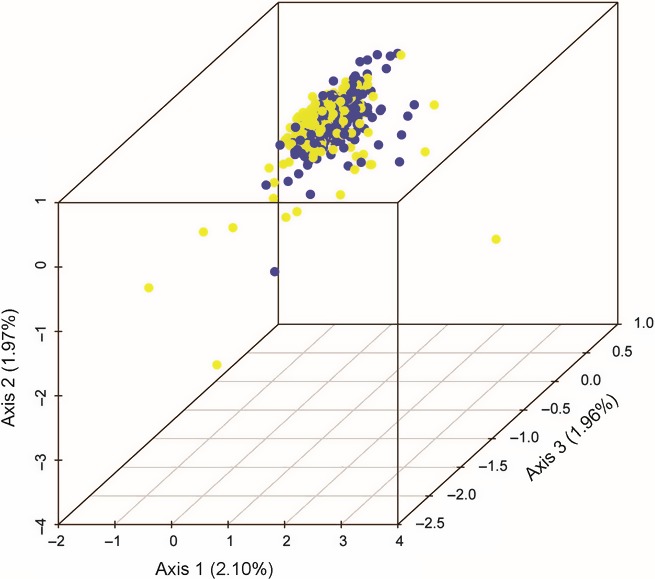
Factorial Correspondence Analysis plot of categorical data exploring correspondence between individuals and alleles. Tight clustering of individuals and alleles indicates low diversity and a lack of population structure. The initial sampling effort is indicated in yellow, while the second sampling effort is indicated in blue

As in Bayesian clustering analyses, we found no indication of separate clustering of individuals based on shared alleles in the FCA analysis. FCA results (Figure 4) show a single tight cluster; thus, there no significant pattern of allelic distributions among individuals.

### 3.3 Demographic expansion

Neutrality tests of mtDNA sequences in arlequin were both negative; however, Tajima's *D* (=−0.80; *p *=* *0.23) was not significantly different from zero while Fu's *F*_*S*_ (=−13.60; *p *=* *0.01) was significant. This is not unexpected, as Tajima's *D* has been shown to lack power in analyses of population growth (Ramos‐Onsins & Rozas, 2002). The mismatch distribution was unimodal (Appendix S2) and not significant; thus, the model of rapid demographic (SSD = 0.01, *p *=* *0.14; τ = 3.34, 95% CI = 1.04–5.68) and spatial (SSD = 0.01, *p *=* *0.31; τ = 3.03, CI = 0.80–4.70) expansion could not be rejected. The “starburst” pattern observed in the mtDNA network (Figure 2), with all neighboring haplotypes differing by just one to two nucleotides, is also consistent with a model of population growth. Treating all genotypes as representing a single population, we estimated the imbalance index β = 4.53 (range = 1.55–16.50), consistent with a model of population growth (β > 1).

## 4 DISCUSSION

### 4.1 Molecular demography

*Desmodus rotundus* from north eastern Mexico are characterized by a shallow evolutionary history and low genetic diversity with evidence of rapid population expansion. The starburst pattern of the mtDNA network is characteristic of rapid expansion evidenced by only a few founder haplotypes, followed by the recent evolution of closely related haplotypes (Slatkin & Hudson, 1991). This demographic model is further supported by a significantly negative Fu's *F*_S_, mismatch distribution analyses, and estimates of Kimmel's β > 1, showing significant recent population growth.

The control region of the mitochondrial DNA genome is known to accumulate mutations more rapidly than cytochrome *b* (Meyer, 1993; Parsons et al., 1997). With only 34 haplotypes (Figure 2) and 0.5% average pairwise sequence divergence across 602 sampled individuals, these results strongly contrast with the much higher diversity reported in earlier studies that investigated the more slowly evolving cytochrome *b* gene (cyt *b*) in populations from the core of the species’ range. In comparison, Ditchfield (2000) sequenced 300 bp of cyt *b* for 7 *D. rotundus* from coastal Brazil and found 3.2% sequence divergence. Martins et al. (2007) sequenced 832 bp of cyt *b* from 50 *D. rotundus* from Brazil, French Guyana, and Costa Rica. They recovered 30 unique haplotypes differentiated at 175 segregating sites, with genetic structure among five major ecodomains. Later, Martins et al. (2009) added to their previous sampling effort and found 72 cyt *b* haplotypes from 118 individuals. In this study, they found 233 segregating sites across 832 bp (=28%) as compared to our 14 segregating sites across a smaller fragment size (=3.2% divergence). Finally, Pinto (2009) sampled populations of *D. rotundus* from either side of the Andes in Ecuador and sequenced the entire cyt *b* gene (1140 bp). From 134 individuals that were sampled, there were 22 haplotypes with 147 segregating sites. The samples in these previous studies were collected across the core of the range of *D. rotundus* (South America into southern Mexico), rather than on the edge of the species’ distribution as in our study. Although we also employ a different mtDNA locus, it is evident that populations of *D. rotundus* from more southerly core portions of their range have higher genetic diversity than occurs at the northern leading edge of their range.

Our sampling, although localized to the northern part of the range in Mexico, covered a large area of approximately 46,000 square kilometers. Interestingly, previous mtDNA sequence analyses of *D. rotundus* (Martins et al., 2007, 2009) found low sequence divergence among localities separated by over 400 km, but also found larger genetic differences over short distances. Instead of simple isolation by distance, these authors found low sequence divergence within an ecoregion and large divergences between ecoregions. Therefore, samples collected across short distances but in different ecoregions showed high divergences. Our samples were collected in a larger area than some of the ecoregions described by Martins et al. (2007, 2009) and spanned multiple ecoregions in Mexico (Olson et al., 2001), yet we found extremely low mtDNA sequence divergence across our entire study area. This pattern is explained by the leading‐edge model where broadly distributed species harbor more genetic diversity in the core of their range (e.g., Ditchfield, 2000; Martins et al., 2007, 2009) than in the leading edge of their range where diversity is expected to be influenced by range contractions and expansions, founder events, and resulting population bottlenecks (Hewitt, 1993, 1996, 1999; Hewitt 2004).

Romero‐Nava et al. (2014) used a subset of seven of 12 microsatellite loci used in the current study to analyze the genetic diversity of *D. rotundus* from 15 states across Mexico. One locality overlapped our sampling area, where they collected 12 bats from Ciudad Valles, San Luis Potosí (Figure 1). They found notably low levels of allelic richness and genetic diversity at this location relative to the others sampled. Romero‐Nava et al. (2014) found moderate levels of homozygotes per locus among the individuals genotyped (Table 2), while we found higher numbers of homozygotes (Table 2) with much larger sample sizes in this region. Romero‐Nava et al. (2014) concluded that recent gene flow among their sampling localities was limited, while our analyses strongly support panmixia. Their sampling was from sites that had large unsampled areas between them and sample sizes per site ranged from 9 to 15, which limited the power of their inferences across Mexico. Two microsatellite loci in our data had excess homozygotes across samples. Both of these loci were used in Romero‐Nava et al. (2014) and were not found to have excessive homozygosity or null alleles. There are other explanations for the demographic results such as selection or a bias driven by our sampling scheme (Grant, 2015). However, examining the mtDNA sequences and microsatellite data reveals that there is a lack of diversity at both markers and loss of intermediate‐sized alleles (Appendix S1), which are more likely due to rapid demographic expansion.

**Table 2:** The number of homozygotes per locus, total samples per locus, and the proportion of homozygotes to total samples for Romero‐Nava et al. (2014) and this study

Locus	HO^a^	HO/T^a^	T^a^	HO^b^	HO/T^b^	T^b^
B10	15	0.37	41	381	0.63	602
B11	14	0.48	29	314	0.52	602
D06	17	0.41	41	381	0.63	602
G10	17	0.39	44	258	0.43	602
H02	11	0.28	39	452	0.75	602
C11	10	0.24	41	237	0.39	602
D02	8	0.20	41	172	0.29	602

HO, Homozygotes; T, total samples; HO/T, proportion of homozygotes at the locus across all samples.

a Romero‐Nava et al., 2014.

b This study.

## 5 CONCLUSIONS

Our results demonstrate strong signals of population expansion in the north eastern portion of the range of *D. rotundus*. This interpretation of our data is supported by panmixia with low genetic diversity, low population differentiation, loss of intermediate‐sized alleles at microsatellite loci, and very low mtDNA haplotype diversity with all haplotypes being very closely related. To precisely estimate the timing and direction of this expansion, a phylogeographic study from across the range of *D. rotundus* should be conducted in order to elucidate evolutionary forces affecting populations across the species’ range. Such a study might also reveal whether or not *D. rotundus* populations have been altered through the large‐scale introduction of domestic livestock from Europe in the 15th and 16th centuries.

Fossil evidence demonstrates that *D. rotundus* previously ranged considerably further north, and, given their current proximity to the U.S. border, climate change modeling paired with species distribution modeling suggests that this species could expand their range northward into what is now the U.S. (Hayes & Piaggio, in review). As areas north of the current distribution of *D. rotundus* become more suitable for their physiological needs, it is likely that they will colonize those areas and transport their rabies virus variants to new regions and newly susceptible hosts.

## AUTHOR CONTRIBUTIONS

A.J.P., A.E.T., G.F.M., A.M‐V., and L.L. conceived the study and/or study design; I.A.O., A.E.T., and A.J.R. collected samples and data; A.J.P., A.L.R., J.W.F., A.E.T., and J.L.N. analyzed the data; and A.J.P. led the writing.

## CONFLICT OF INTEREST

None declared.
