# Supplementary material for: Genetic demography at the leading edge of the distribution of a rabies virus vector
Source: Ecol Evol. 2017 Jun 9;7(14):5343–51. doi: 10.1002/ece3.3087 (PMC5528231; doi:10.1002/ece3.3087)
Supplement: Supplementary file 3 [file ECE3-7-5343-s003.pdf]

Allele Frequencies with Graphs by Population and Locus for Codominant Data

**Data Sheet**	msats final no coastal s Me
**Data Title**	GMConvert output file WITH modifications (6/7/13) - FINAL DATASET!!!

**No. Loci**	12
**No. Samples**	602
**No. Pops.**	1

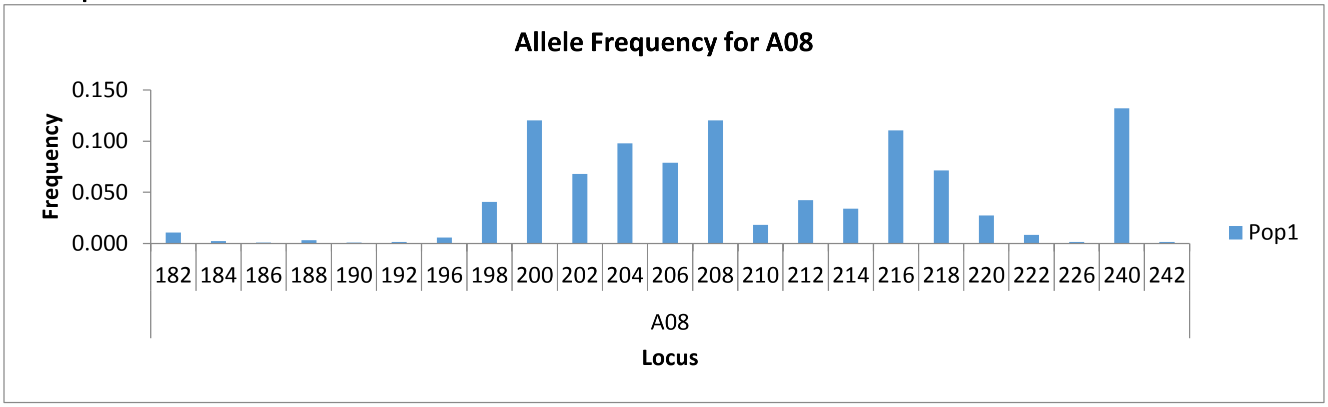

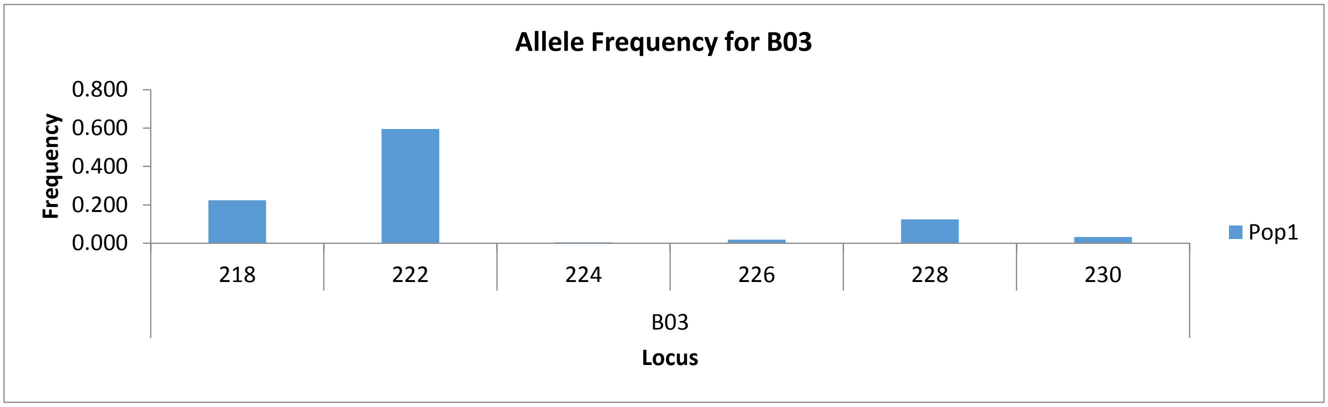

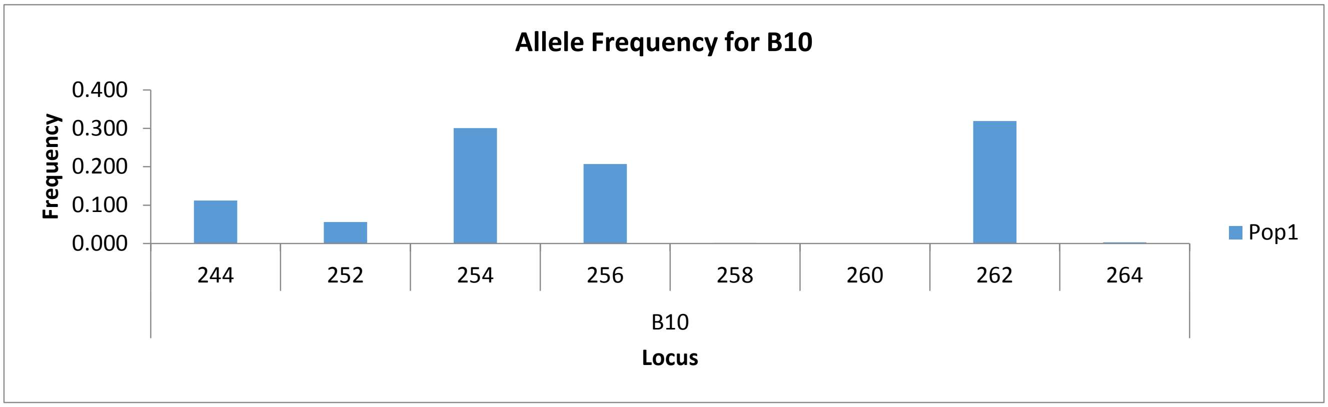

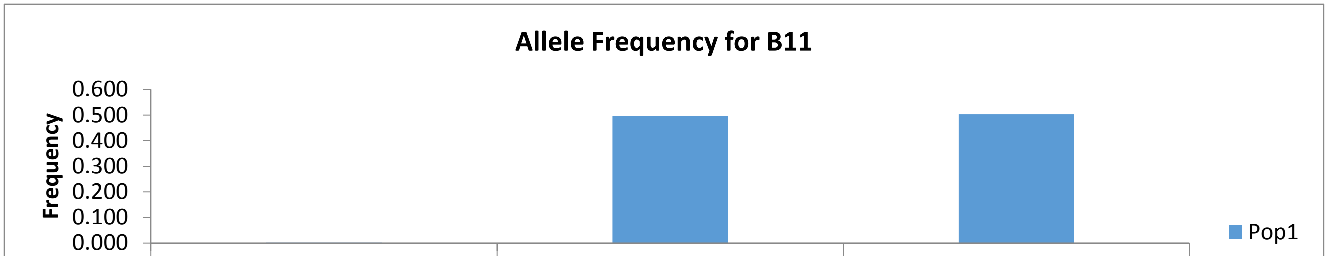

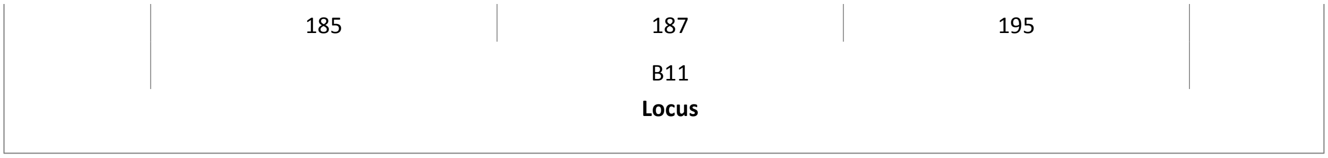
185
187
195
B11
Locus

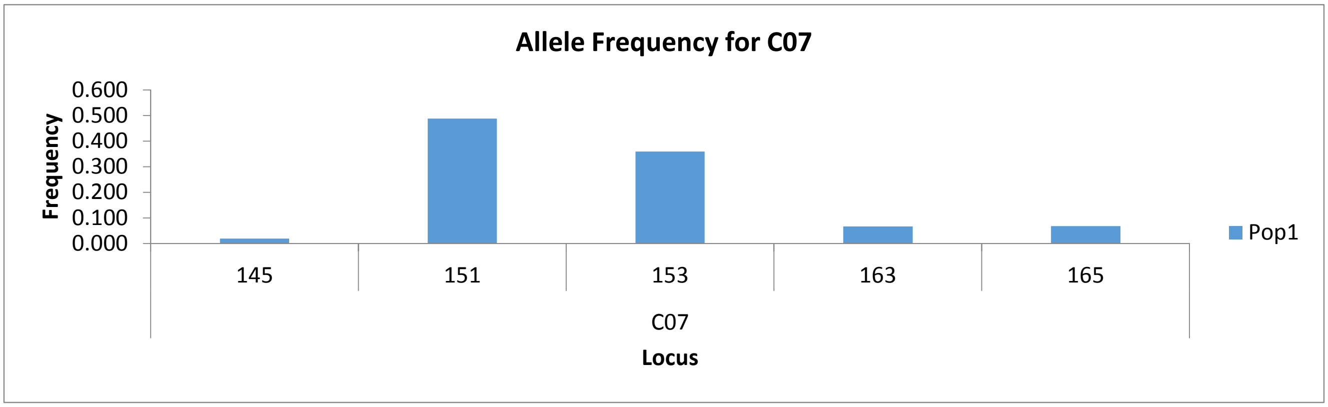
Allele Frequency for C07
Frequency
0.600
0.500
0.400
0.300
0.200
0.100
0.000
145
151
153
163
165
C07
Locus
Pop1

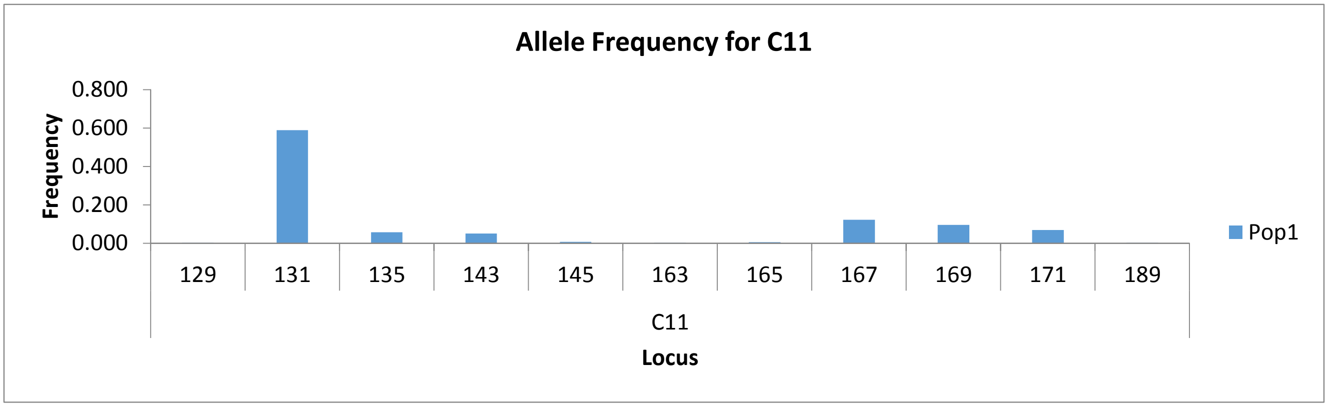
Allele Frequency for C11
Frequency
0.800
0.600
0.400
0.200
0.000
129
131
135
143
145
163
165
167
169
171
189
C11
Locus
Pop1

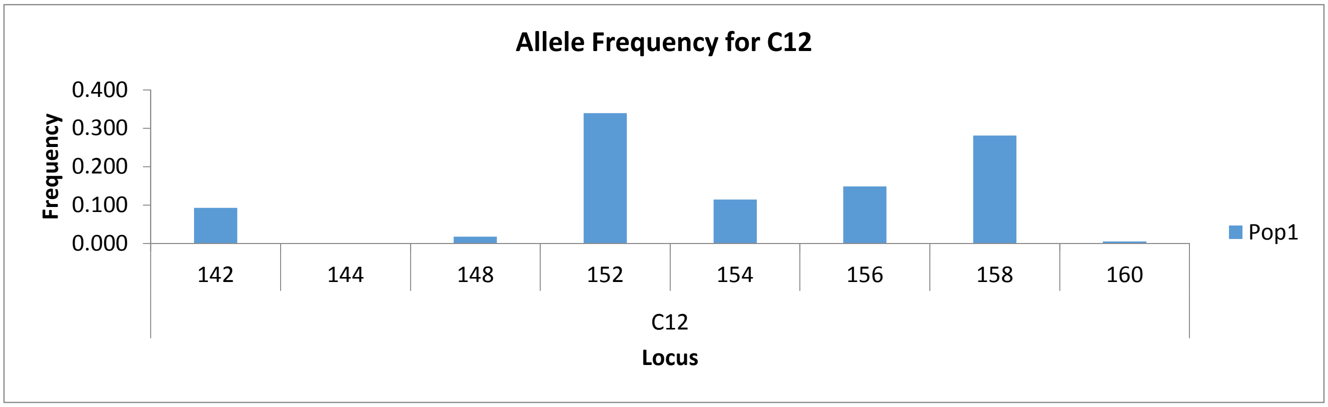
Allele Frequency for C12
Frequency
0.400
0.300
0.200
0.100
0.000
142
144
148
152
154
156
158
160
C12
Locus
Pop1

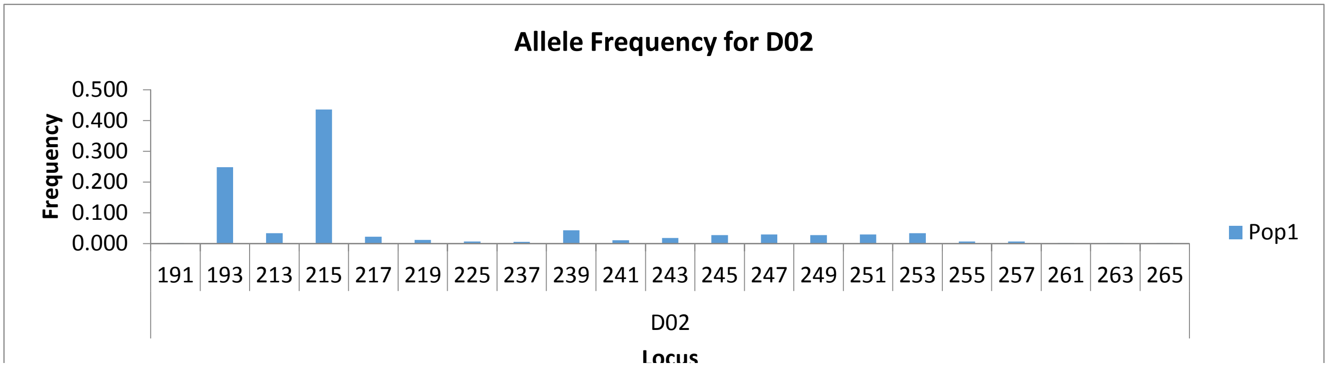
Allele Frequency for D02
Frequency
0.500
0.400
0.300
0.200
0.100
0.000
191
193
213
215
217
219
225
237
239
241
243
245
247
249
251
253
255
257
261
263
265
D02
Pop1

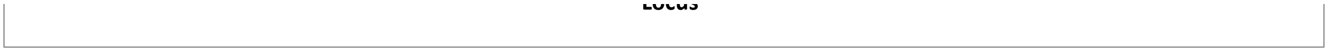
Locus

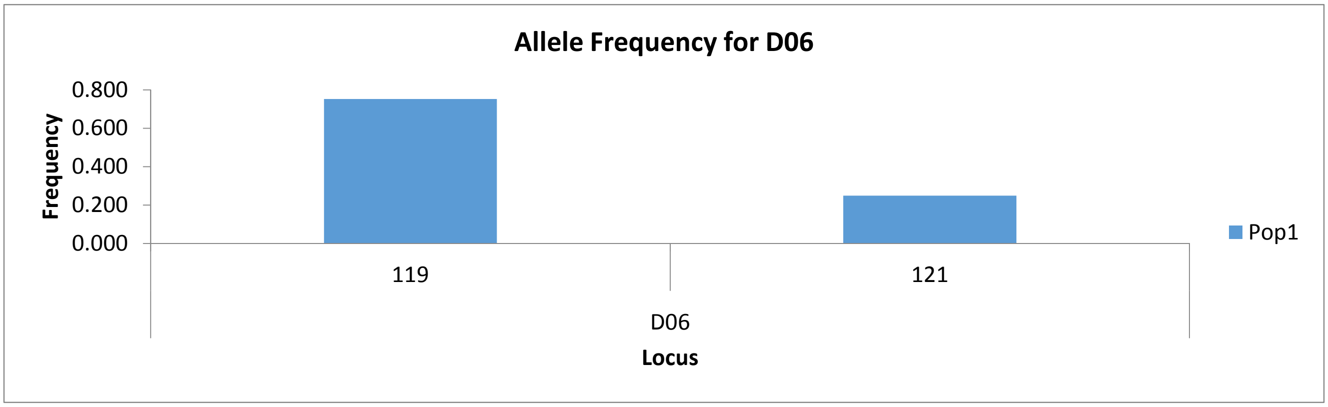
Allele Frequency for D06
Frequency
0.800
0.600
0.400
0.200
0.000
119
121
D06
Locus
Pop1

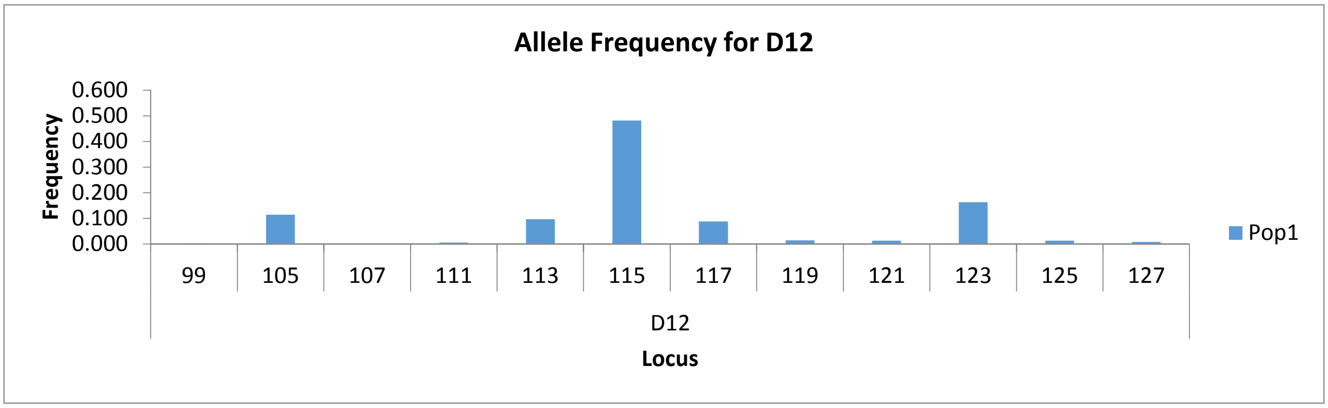
Allele Frequency for D12
Frequency
0.600
0.500
0.400
0.300
0.200
0.100
0.000
99
105
107
111
113
115
117
119
121
123
125
127
D12
Locus
Pop1

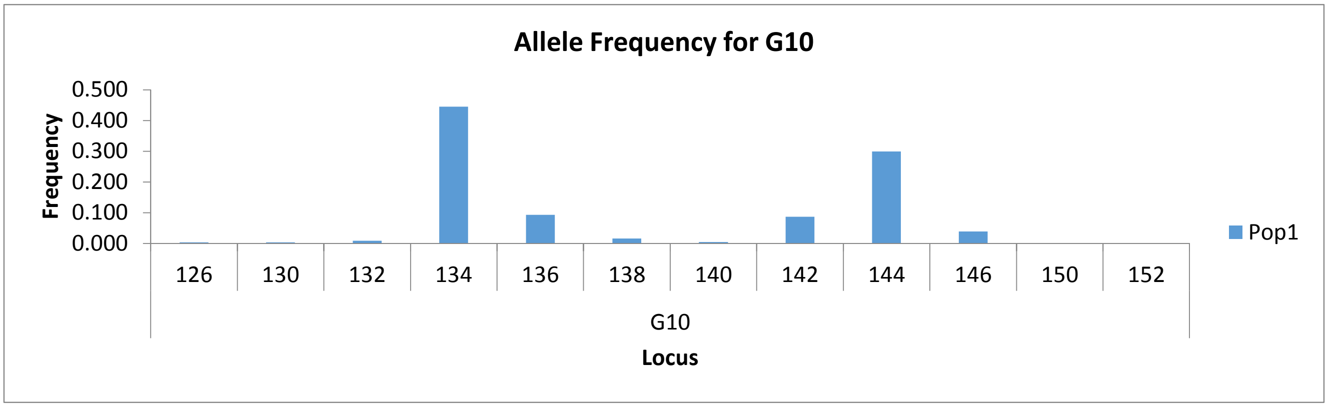
Allele Frequency for G10
Frequency
0.500
0.400
0.300
0.200
0.100
0.000
126
130
132
134
136
138
140
142
144
146
150
152
G10
Locus
Pop1

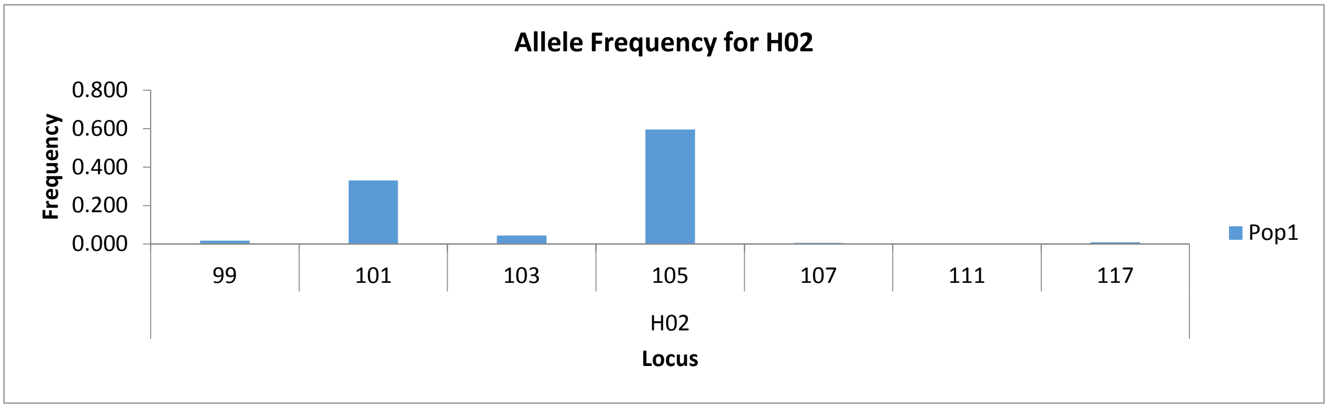
Allele Frequency for H02
Frequency
0.800
0.600
0.400
0.200
0.000
99
101
103
105
107
111
117
H02
Locus
Pop1

Allele Frequencies by Populations For Graph By Locus

Locus	Allele	Pop1
A08	182	0.011
	184	0.002
	186	0.001
	188	0.003
	190	0.001
	192	0.002
	196	0.006
	198	0.041
	200	0.120
	202	0.068
	204	0.098
	206	0.079
	208	0.120
	210	0.018
	212	0.042
	214	0.034
	216	0.110
	218	0.071
	220	0.027
	222	0.008
	226	0.002
	240	0.132
	242	0.002

Locus	Allele	Pop1
**B03**	**218**	0.224
	**222**	0.595
	**224**	0.005
	**226**	0.019
	**228**	0.125
	**230**	0.032

Locus	Allele	Pop1
**B10**	**244**	0.112
	**252**	0.056
	**254**	0.301
	**256**	0.207
	**258**	0.002
	**260**	0.001
	**262**	0.319
	**264**	0.003

Locus	Allele	Pop1
**B11**	**185**	0.002
	**187**	0.495
	**195**	0.503

Locus	Allele	Pop1
**C07**	**145**	0.019
	**151**	0.488
	**153**	0.359
	**163**	0.066
	**165**	0.068

Locus	Allele	Pop1
**C11**	**129**	0.002
	**131**	0.590
	**135**	0.056
	**143**	0.051
	**145**	0.007
	**163**	0.002
	**165**	0.006
	**167**	0.122
	**169**	0.095
	**171**	0.068
	**189**	0.002

Locus	Allele	Pop1
**C12**	**142**	0.092
	**144**	0.001
	**148**	0.017
	**152**	0.339
	**154**	0.115
	**156**	0.149

	**158**	0.281
	**160**	0.005

**Locus**	**Allele**	**Pop1**
**D02**	**191**	0.001
	**193**	0.248
	**213**	0.034
	**215**	0.436
	**217**	0.022
	**219**	0.012
	**225**	0.007
	**237**	0.006
	**239**	0.043
	**241**	0.011
	**243**	0.017
	**245**	0.027
	**247**	0.029
	**249**	0.027
	**251**	0.029
	**253**	0.034
	**255**	0.007
	**257**	0.007
	**261**	0.001
	**263**	0.001
	**265**	0.001

**Locus**	**Allele**	**Pop1**
**D06**	**119**	0.752
	**121**	0.248

**Locus**	**Allele**	**Pop1**
**D12**	**99**	0.002
	**105**	0.115
	**107**	0.001
	**111**	0.006
	**113**	0.096
	**115**	0.482
	**117**	0.087
	**119**	0.014
	**121**	0.013
	**123**	0.163
	**125**	0.013
	**127**	0.007

**Locus**	**Allele**	**Pop1**
**G10**	**126**	0.003
	**130**	0.003
	**132**	0.008
	**134**	0.445
	**136**	0.093
	**138**	0.016

	**140**	0.004
	**142**	0.086
	**144**	0.299
	**146**	0.039
	**150**	0.001
	**152**	0.002

**Locus**	**Allele**	**Pop1**
**H02**	**99**	0.017
	**101**	0.330
	**103**	0.044
	**105**	0.596
	**107**	0.005
	**111**	0.001
	**117**	0.008
